# S04-2 More than a club' - experiences gained by piloting the Sport Clubs for Health approach in Hungary

**DOI:** 10.1093/eurpub/ckac093.018

**Published:** 2022-08-29

**Authors:** Reka Veress

**Affiliations:** University and Leisure Sport Federation, National School, Budapest, Hungary

**Keywords:** SCforH, sport clubs, pilot project, HEPA, Hungary

## Abstract

**Issue/problem:**

According to the latest Eurobarometer, only 5% of the active population in Hungary are sport club members. The “Sport Clubs for Health” (SCforH) approach was developed to help unlock the health potential of sports clubs and increase the offer of recreational sport programmes. The SCforH guidelines have been recognized in 2013 by the EU, as one of the indicators of the Council recommendations on promoting HEPA across sectors.

**Description of the problem:**

The Hungarian Ministry of Human Capacities has recognized the need to support the implementation of SCforH approach, and decided to finance the “More than a club” pilot project. This project aimed to develop complex services that enable local and regional sports clubs to build their capacities and provide regular, health-focused physical activities supervised by sport professionals. Within the project, we tested and analysed what kind of organizational and service development measures are needed to be able to provide more health-focused programmes to the population, within sports clubs.

**Results (effects/changes):**

We managed to recruit 190 participants (drop-out rate = 12.7%) and involve them for at least one year into the following sport activities in a multisport environment: table tennis; zumba; Nordic walking; cycling; gymnastics; spine training and breathing technics; and ‘father-son' football. The programme is ongoing, and participants are monitored regularly in relation with their health-status and attitude towards physical activities. The adherence to the programme is 87.3%.

**Lessons:**

This project showed this type of a SCforH intervention may be feasible for implementation at a larger scale in multisport clubs. The further aim of the project is to prepare and test the Hungarian model of health-oriented sports clubs, by the end of April 2021. Based on the lessons learned from this pilot project, a long-term goal is to implement this kind of a SCforH intervention in several large Hungarian sports organizations and contribute to improving health at the population level.

**Main messages:**

The implementation of SCforH guidelines can be realized in a gradual manner with dedicated leadership. Top-sport oriented clubs possess the capacity to implement the SCforH approach, but incentives and training are needed to facilitate the implementation.

